# *yti*B and *yth*A Genes Reduce the Uranium Removal Capacity of *Bacillus atrophaeus*

**DOI:** 10.3390/ijms20071766

**Published:** 2019-04-10

**Authors:** Li Wang, Shiqi Xiao, Xiaoming Chen, Shilin Chen, Shanqiang Wang, Chao Wang, Yunlai Tang, Faqin Dong

**Affiliations:** 1 School of Life Science and Engineering, Southwest University of Science and Technology, Mianyang 621010, China; wangli@swun.edu.cn (L.W.); m18381654985_1@163.com (S.X.); w1870835752@sina.com (C.W.); tyl@swust.edu.cn (Y.T.); fqdong@swust.edu.cn (F.D.); 2 College of Life Science and Technology, Southwest Minzu University, Chengdu 610041, China; 3 Department of Bioengineering, University of California at Berkeley, CA 94703, USA; alexchensl@berkeley.edu; 4 State Key Laboratory of NBC Protection for Civilian of China, Beijing 102205, China; 13651135504@163.com

**Keywords:** *Bacillus atrophaeus*, gene *yth*A, Gene *yti*B, microbial remediation, uranium contamination

## Abstract

Two *Bacillus atrophaeus* strains, the first being a highly stress-resistant ATCC 9372 strain and the Ua strain identified from a chromium mine by our lab, differ in their abilities to tolerate and remove Uranium (VI) from contaminated water. An increase in U(VI) concentration in growth media led to a decrease in the tolerance and bio-remedial capacity of both strains. However, under high concentrations of U(VI) in the growth media, the ATCC 9372 strain demonstrated a higher tolerance and a higher removal capacity than the Ua strain. Two approaches, transcriptome sequencing and transgenic technology, were used to elucidate the relationship between particular genes within these two strains and their U(VI) removal capacity. Sequencing confirmed the expression of two genes unique to the Ua strain, previously designated *yti*B and *yth*A. They encode putative proteins that show the highest levels of identity to carbonic anhydrase and cytochrome bd terminal oxidase I, respectively. Using the pBE-S DNA vector, *yti*B and *yth*A were transformed into the ATCC 9372 strain of *Bacillus atrophaeus*. Under a U(VI) concentration of 120 mg/L, the removal rates of the transgenic ATCC 9372-*yti*B and ATCC 9372-*yth*A strains decreased by 7.55% and 7.43%, respectively, compared to the removal rate of the control strain transformed with empty plasmid. The results suggest that both *yth*A and *yti*B genes have a negative influence on the uranium removing capacity of *Bacillus atrophaeus*. This finding will help to elucidate the molecular mechanisms of uranium removal by bacteria.

## 1. Introduction

Uranium is an important strategic and economic resource. Through the development of nuclear industry, nuclear technologies have expanded and are now used extensively in a variety of industrial productions, agriculture, energy production, military, transportation, and health care. Therefore, uranium contamination of soil and water has increased, resulting in serious human and animal diseases and an increased number of deaths [1]. Bioremediation was proposed as a plausible method for the removal of uranium contamination [2,3]. Microbial remediation is a promising technique for bioremediation. In this method microbes are used to either adsorb uranium or change the uranium oxidation state [4,5,6]. Differing abilities for microbial remediation of uranium pollution can be observed even among closely related strains of the same microbial species. The most likely explanation for this phenomenon would be the presence of unique gene(s) within the microbial genomes of particular strains. Therefore, revealing the differences between gene sets of closely related strains is an important way to understand the molecular mechanisms of bioremediation.

There are many methods that can be applied to reveal the differences between two organisms at the DNA, RNA or protein levels. Among them are the metagenomics library-based technique [7], gene chip technology [8], in-situ hybridization [9,10], western blot hybridization and transcriptome sequencing [11,12]. These methods provide high accuracy and high throughput, but there are also limitations specific to each method in relation to their practical application. The novel high-throughput deep sequencing technology has dramatically expanded the kinds of questions that can be answered and for strategies applied to study complex transcriptomes. This approach relies on the generation of featured EST tags corresponding to the fragments of the transcripts in a sample and their subsequent concatenation prior to cloning and sequencing [13]. Transcriptome analysis provides the ability to not only quantify the changes in expression levels for the same genes in different samples, but also to annotate the transcriptome and determine the structure of genes in the genome [14,15]. RNA sequencing has been exploited to analyze the dynamic transcriptome and abundance of specific bacterial genes [16,17,18].

The bacterial strain *Bacillus subtilis* var *niger* was re-classified as the ATCC 9372 strain of *Bacillus atrophaeus* by Nakamura in 1989 [19]. This strain can tolerate various adverse environments, such as high temperature, ultraviolet ionizing radiations, and toxic chemicals. Another strain of *Bacillus atrophaeus* found in a chromium mine and designated as Ua strain by our lab [20], is very similar to the ATCC 9372 strain, but it differs in its ability to tolerate and remove Uranium (VI). We proposed earlier that the possible reason for this phenomenon may be due to genetic differences between the strains. In our preliminary work, Pulsed Field Gel Electrophoresis (PFGE) was used to reveal differences between the genomes of *Bacillus atrophaeus* ATCC 9372 and Ua strains. DNA was digested by a combination of *EcoR*I and *Hind*III endonucleases, and a single DNA band that differed between the two strains was identified by PFGE. Sequencing of the DNA fragment isolated from this band revealed that the 3,128 bp long sequence contained *yth*A and *yti*B, genes, that were unique to the Ua strain [20].

In this work, transcriptome sequencing was used to verify gene sequences and to confirm that *yti*B and *yth*A genes are expressed only in the Ua strain. Furthermore, a transgenic approach was applied to demonstrate the influence of these genes on the uranium removal capacity of *Bacillus atrophaeus*. The findings of this work will benefit further studies directed towards an understanding of the molecular mechanisms of uranium removal.

## 2. Results and Discussion

### 2.1. Tolerance of ATCC 9372 and Ua Strains to Different Concentrations of U(VI)

**Figure 1:**
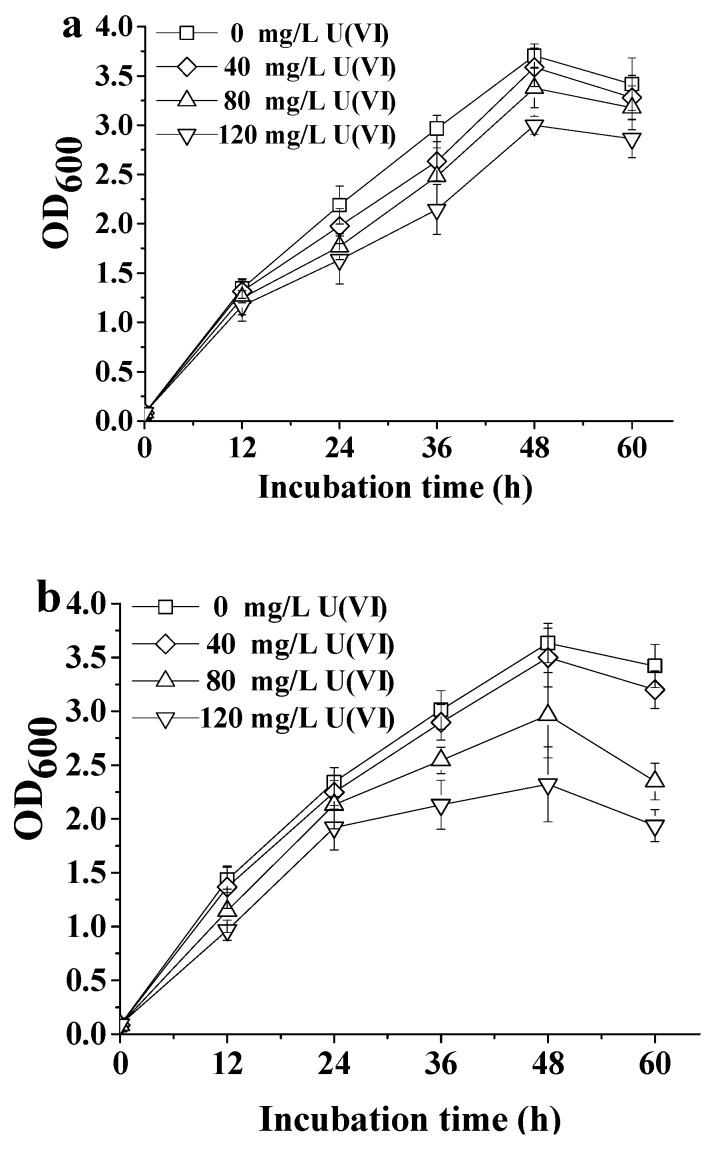
Growth dynamics of the ATCC9372 strain (**a**) and the Ua strain (**b**) in the U(VI) having growth media with different concentrations of uranium. All experiments were performed in triplicate. Average results of tree experiments were shown; bars represent standard deviations.

The growth curves of ATCC 9372 and Ua strains in TGY media containing different concentrations of U(VI) were shown in Figure 1. Better growth of ATCC 9372 strain compared to Ua strain in the medium containing the same U(VI) concentration demonstrated that this strain has a higher U(VI) tolerance than the Ua strain. The growth curve of ATCC 9372 strain reached its maximum OD_600_ value on 48 h after inoculation, and there was no significant change in ATCC 9372 growth dynamics in medium containing 80 mg L^−1^ U(VI) compared to control medium having no uranium. However, a clear decrease in OD_600_ value was observed when the concentration of U(VI) in the growth medium was increased to 120 mg L^−1^. Similarly, the growth curve of the Ua strain reached its maximum 48 h after inoculation, and this strain had a high tolerance to uranium under 40 mg L^−1^ U(VI). Increasing U(VI) concentration led to the decrease in growth rate for both strains; the highest OD_600_ values reached for ATCC 9372 and Ua strains (after 48 h of growth) decreased from 3.70 to 2.99 and 3.63 to 2.32, respectively.

**Figure 2:**
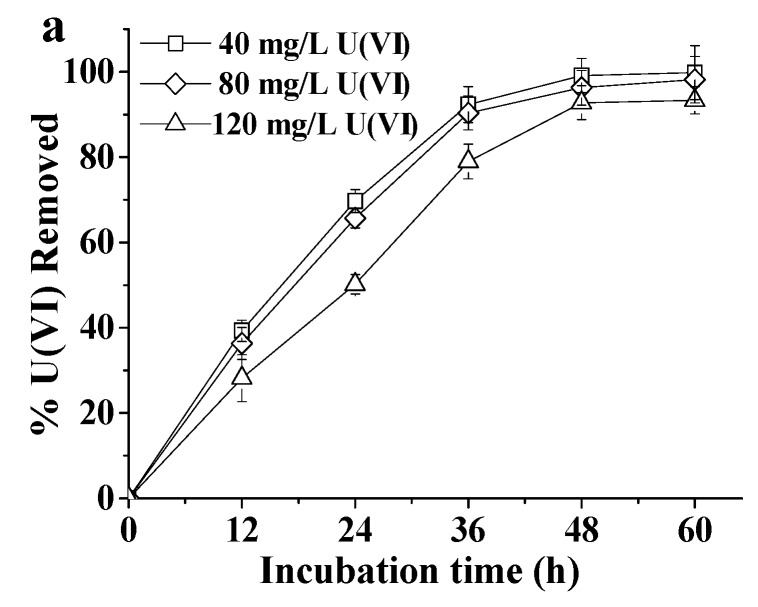

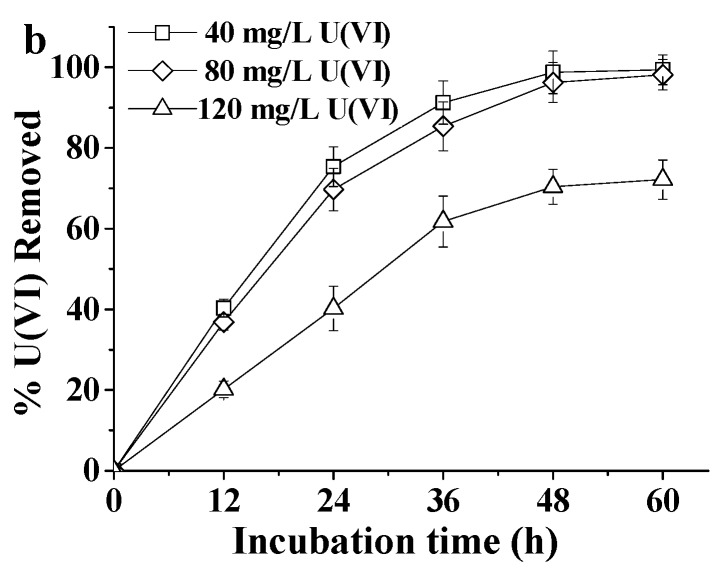
The U(VI) removal capacities of the ATCC 9372 strain (**a**) and the Ua strain (**b**). All experiments were performed in triplicate. The average results of three experiments were shown; bars represent standard deviations.

The removal capacities of U(VI) by ATCC 9372 and Ua strains were shown in Figure 2. At 40 and 80 mg L^−1^ U(VI) the removal capacities of both strains were similar, reaching about 99% in 60 h. However, at 120 mg L^−1^ U(VI) the removal capacities of ATCC 9372 and Ua strains decreased to 93.33% and 72.18%, respectively. In other words, the obtained results revealed that at high U(VI) concentrations the U(VI) removal capacity of ATCC 9372 strain was higher than that of the Ua strain.

### 2.2. Comparison of Bacillus atrophaeus Transcriptomes under Control Conditions and under Exposure to Uranium

Raw RNA sequence reads were generated using Illumina HiSeq2500 equipment. After adapter clipping and quality trimming, clean reads were obtained for pools of total RNAs isolated from *Bacillus atrophaeus* ATCC 9372 and Ua strains, respectively. These clean reads were used in *de novo* assembly. The obtained contigs were assembled into unigenes after the splicing of sequences and the removing of redundancies. All clean reads for each group were compared to the genome of *Bacillus atrophaeus* 1942 strain (http://www.ncbi.nlm.nih.gov/nuccore/310867486?report=fasta) (Table 1). In general, the ratio of mapping of all clean reads should show over 60% similarity with other strains of the same species. If the ratio is beyond 70%, the two samples are considered to have high homology [21]. As shown in Table 1, both *Bacillus atrophaeus* strains, ATCC 9372 and Ua, showed high homology with *Bacillus atrophaeus* 1942. The unigenes from control and experimental samples were annotated by Gene Ontology (GO). All unigenes were divided into three main categories: (i) molecular functions, (ii) cellular components and (iii) biological processes. These three main categories in turn comprised more than 40 subcategories. In the biological process category, the single-organism and cellular processes were the most represented subcategories.

**Figure 3:**
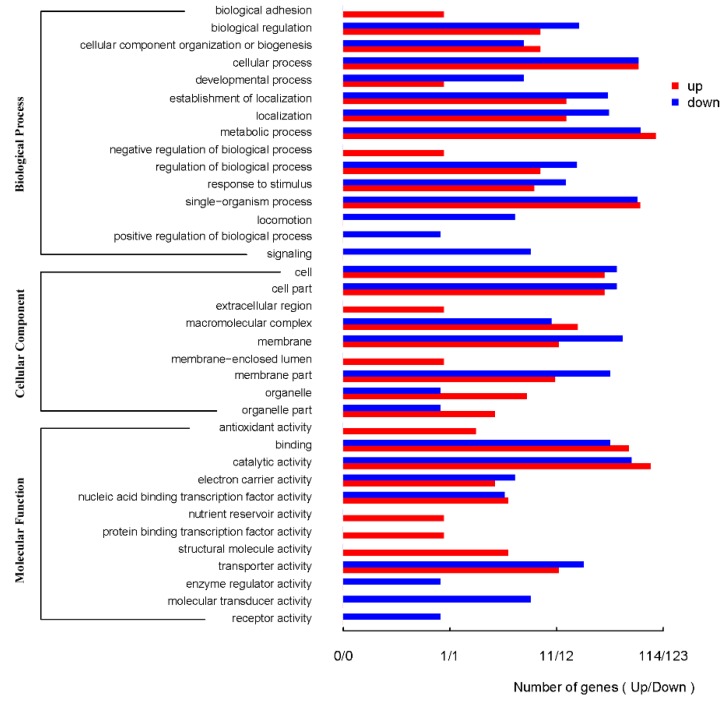
The GO annotations of differentially expressed genes of *Bacillus atrophaeus* ATCC 9372.

**Figure 4:**
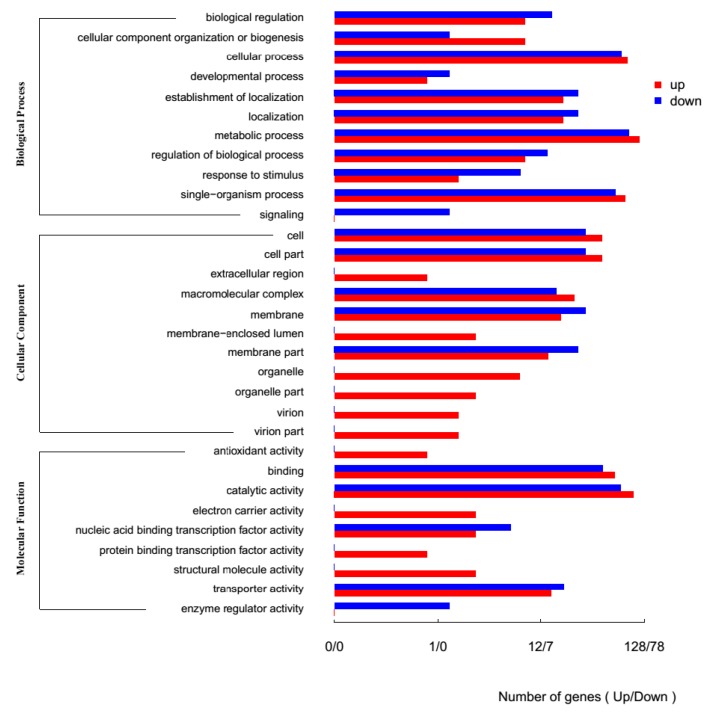
The GO annotations of differentially expressed genes of *Bacillus atrophaeus* Ua.

Differentially expressed genes were analyzed using Kyoto Encyclopedia of Genes and Genomes (KEGG). A total of 8372 differentially expressed unigenes were acquired through comparisons between control gene expression and expression when exposed to 40mg/L U(VI). The comparison revealed 197 up-regulated and 196 down-regulated genes in the ATCC 9372 strain. However, 3793 genes did not show obvious changes in expression levels. 372 unigenes showed significant differential expression between the control and U(VI) groups of genes in the Ua strain, from which 228 genes were up-regulated and 144 genes were down-regulated. Analysis of these differentially expressed unigenes using GO database revealed that most of the regulated genes belonged to metabolic processes. This group of genes was followed by genes involved in catalytic activities, single-organism processes and proteolysis (Figure 3 and Figure 4).

Both ATCC 9372 and Ua strains belong to the same bacterial species of *Bacillus atrophaeus*, and therefore they are expected to have very similar genomic structures. However, their ability to tolerate high concentrations of uranium and their respective capacities to remove U(VI) from the growth media are distinctive. A difference in the gene sets contained within the genomes of the two strains is the most probable reason for their different phenotypes. Therefore, our first task was to identify a predicted small difference between the genomes of the two strains. Currently many methods exist to identify such differences through analysis of genomic DNA and/or sets of transcripts [17]. Most of these methods are expensive and/or need advanced operational skills. We selected the PFGE method to analyze the bacterial genomic DNA because it is relatively easy and cheap. PFGE is widely used for chromosomal DNA separation and electrophoretic karyotyping [22,23,24]. For analysis and comparison of the two bacterial transcriptomes, we applied high-throughput sequencing. RNA sequencing (RNA-seq) is a powerful tool for dissecting the relationships between genotypes and phenotypes, and a way to find differences between two transcriptomes. Free of many of the limitations that characterize other transcriptomic approaches, such as microarray and tag-based sequencing, RNA-seq has significantly changed the speed and accuracy of protokaryotic transcriptome analysis [16,25].

### 2.3. Isolation and Sequence Analysis of ytiB and ythA Genes

*yti*B and *yth*A genes were identified solely from the transcriptome sequencing results obtained for the Ua strain. In the KEGG database the *yti*B gene was present under the name *Cyn*T (Gene ID 2626) and the *yth*A gene under the name *Cyd*A (Gene ID 2629). In the Non-redundant database, the *yti*B gene was predicted to encode carbonic anhydrase, while the *yth*A was predicted to encode a subunit I of cytochrome bd terminal oxidase.

The nucleotide sequence of *yti*B cDNA was 570 bp (GenBank accession no: MH142257) and contained an open reading frame of 564 bp, encoding a putative protein of 187 amino acids. The deduced molecular weight was 21.1KDa and predicted pI was 6.16. The ytiB protein contained a domain (29–181aa) characteristic to the beta-carbonic anhydrase clade and therefore belonged to the beta-carbonic anhydrase super family. Analysis using SigalIP 4.1 software revealed that the ytiB protein had no signal peptides but also did not belong to the list of non-secreted proteins. The predicted secondary structure of the ytiB protein consisted mainly of alpha helix (48.13%), extended strand (16.58%), beta turn (8.02%) and random coiled sequence (27.27%) structures. The predicted tertiary structure of the ytiB protein was modeled based on the template, which was a crystal structure of the ‘cab’ type beta class carbonic anhydrase from *Methanobacterium thermoautotrophicum* (PDB: 1g5c.1.A). The *ytiB* protein had 37.04% identity to this ‘cab’ type beta class carbonic anhydrase. Phylogenetic analysis revealed that the *ytiB* protein had the closest relationship to the carbonic anhydrase from *Bacillus atrophaeus*.

The nucleotide sequence of the *yth*A cDNA was 1732 bp (GenBank accession no: MH142256) and contained an open reading frame of 1290 bp, encoding a putative protein of 429 amino acids. The deduced molecular weight of the protein was 47.7 KDa and the predicted pI was 9.06. The *yth*A A protein had a domain named ‘cytochrome bd terminal oxidase subunit I’ (1–419 aa), which belonged to the cytochrome bd terminal oxidase I super family. SigalIP 4.1 showed that the *yth*A protein had no signal peptides and was a non-secreted protein. The predicted secondary structure of the *yth*A protein contained alpha helix structures (49.18%), extended strand (18.65%), beta turn (4.20%) and random coiled sequence (27.97%). The tertiary structure of the *yth*A protein was modeled based on the template of the crystal structure of bd-type quinol oxidase subunit I from *Geobacillus thermodenitrificans* (PDB: 5doq.1.A). The *yth*A protein possessed 43.13% identity to subunit I of the bd-type quinol oxidase. A rectangular phylogenetic tree was produced with the aim to assess the relationship of the *yth*A protein to other known proteins. The *yth* protein belonged to the group of *Bacillus atrophaeus*, with cytochrome bd terminal oxidase subunit I grouped as the closest neighbor.

Our previous study showed that *yti*B and *yth*A genes were discovered only in the genome of the Ua strain [20]. In this study, transcript levels of *yti*B and *yth*A genes were calculated using the RSEM software. The results showed that the expression of both genes in the Ua strain were detectible after 48h treatment with 40mg/L U(VI) (Table 2). The metabolic pathways of *yti*B and *yth*A gene products were analyzed using KEGG databases. It was found that *yti*B was involved in nitrogen metabolism, while *yth*A was involved in oxidative phosphorylation and functioning of the two-component system. Since the metabolic pathways in which *yti*B and *yth*A gene products were involved are very complex and included a large number of different proteins, it was impossible through use of bioinformatics alone to predict the interacting partners of these two proteins, which potentially could be responsible for the removal of uranium by *Bacillus atrophaeus*. Without further experiments, it was difficult at this stage to explain the molecular mechanisms behind the negative influence of *yti*B and *yth*A on uranium removal capacity.

In our preliminary study, PFGE was applied to discover differences in the gene sets of ATCC 9372 and Ua genomes. The results showed that *yti*B and *yth*A genes were present only in the Ua strain [20]. It was possible in our case to use PFGE to find differences between the two gene sets because of the high similarity of the studied organisms (two strains of the same species) and consequent similarity in their genomes. More experimental data will be required to decide if this method can be utilized for species that are more distally related. In addition, we had the good fortune that both genes were situated close to one another in the bacterial genome, on the same relatively large DNA fragment identified by PFGE. If *yti*B and *yth*A were located in different parts of the bacterial genome and after restriction were situated on smaller DNA fragments, genes could be lost during PFGE, since small DNA fragments would quickly run off the gel.

Transcriptome sequencing enabled us to identify cDNA sequences of all transcribed genes and obtain their full-length sequences. The results obtained by transcriptome sequencing basically confirmed the result of PFGE comparison between ATCC 9372 and Ua strains. In that the two different transcribed genes, *yti*B and *yth*A, were present only in the genome of the Ua strain. Transcriptome sequencing also provided corrections to the lengths of *yti*B and *yth*A genes, which, according to the results of the genomic fragment sequencing were predicted to be 570 bp and 1732 bp long, respectively. The full-length sequence of the *yti*B cDNA was found to be 2 bp longer and the *yth*A cDNA was 339 bp longer than the sequences predicted earlier based on genomic data [20]. Our data indicates that both PFGE and transcriptome sequencing were efficient in finding differences between two closely related microbial genomes. The availability of efficient and accurate technologies for gene discovery will be very useful in our future exploration of the molecular mechanisms of bacterial responses to environmental stimuli and stresses.

### 2.4. Influence of ytiB and ythA on the U(VI) Removal Capacity of Bacteria in Transgenics

**Figure 5:**
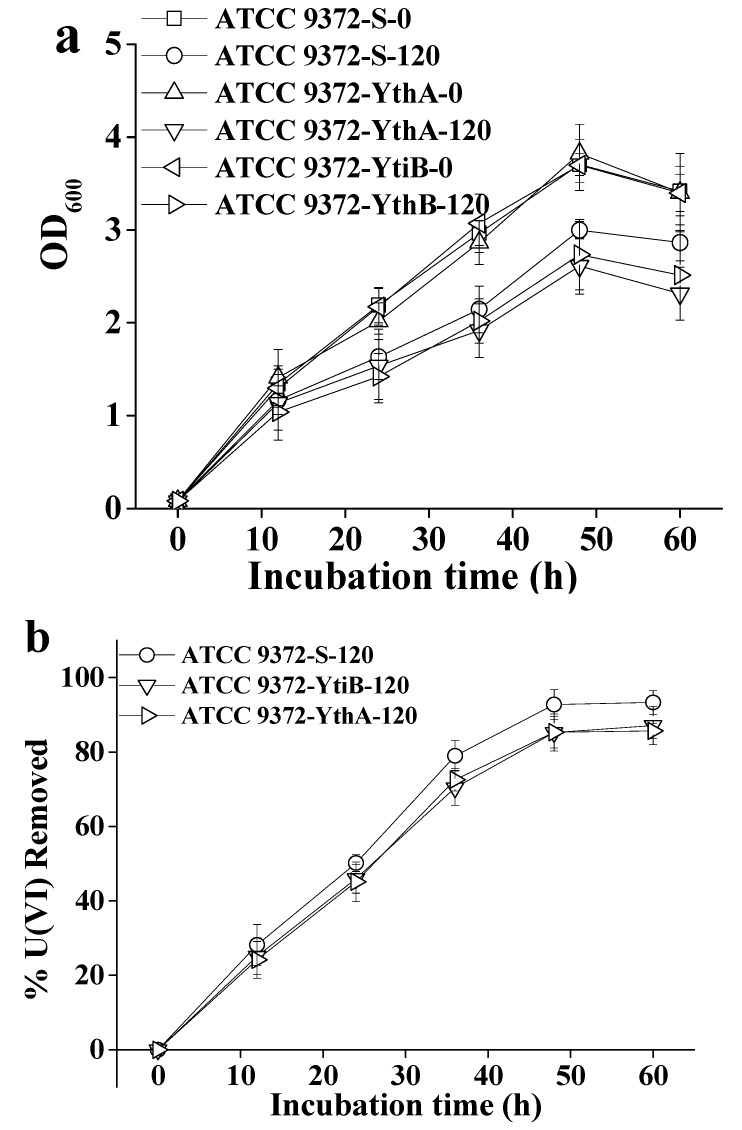
The growth rate (**a**) and U(VI) removal capacity (**b**) of transgenic ATCC 9372 strains in U(VI)-containing LB media. ATCC 9372-S-0—the ATCC 9372 strain transformed with the pBE-S DNA and grown in the media containing no U(VI); ATCC 9372-S-120—the ATCC 9372 strain transformed with the pBE-S DNA and grown in the media containing 120 mg L^−1^ U(VI); ATCC 9372-*yti*B-0—the ATCC 9372 strain transformed with the *yti*B gene and grown in the media containing no U(VI); ATCC 9372-*yti*B-120—the ATCC 9372 strain transformed with the *yti*B gene and grown in media containing 120 mg L^−1^ U(VI); ATCC 9372-*yth*A-0—the ATCC 9372 strain transformed with the *yth*A gene grown in the media containing no U(VI); ATCC 9372-*yth*A-120—the ATCC 9372 strain transformed with the *yth*A gene grown in media containing 120 mg L^−1^ U(VI). All experiments were performed in triplicate. The average results for three experiments are shown; bars represent standard deviations between repeats.

The generation of transgenic strains using ATCC 9372 as the recipient strain was carried out as described in Materials and methods. Recombinant expression vectors were verified by restriction enzyme digestion with *Mlu*I and *Xba*I. The growth dynamics of ATCC 9372-*yti*B and ATCC 9372-*yth*A transgenic strains and the ATCC 9372-S negative control strain, as well as their U(VI) removal capacity at 120 mg L^−1^ of U(VI) in the growth media are shown in Figure 5. All experiments were repeated three times, and averaged results were used in the figure.

As is shown in Figure 5, transgenic ATCC 9372-S, ATCC 9372-*ythA* and ATCC 9372-*yti*B strains as well as the wild type ATCC 9372 strain had no obvious differences in growth dynamics in the media containing 0 mg L^−1^ U(VI). These data indicated that the transgenic manipulation did not produce any harmful changes to the original ATCC 9372 strain. The growth rate of ATCC 9372-*yti*B strain decreased at the U(VI) concentration of 120 mg L^−1^. The uranium removal capacity of the ATCC 9372-S strain was equal to the control ATCC 9372 strain and higher than that of the ATCC 9372-*yti*B strain (*t*-test, *p* < 0.05). The U(VI) removal capacity of the ATCC 9372-S strain in the presence of 120 mg L^−1^ U(VI) in the growth media was 92.77% on 48 h after inoculation. In contrast, the removal rate of ATCC 9372-*yti*B strain was 85.22%, which was very similar to the removal rate of the ATCC 9372-*yth*A strain. These results confirmed that the expression of each of the two genes tested has negative effects on the U(VI) removal capacity of the bacteria.

Analysis of gene function can be achieved through the generation of transgenic organisms with (i) expression of a transgene novel to the recipient organism, (ii) overexpression of a gene already existing in the recipient organism, or (iii) by application of gene knockout technologies [26,27]. In this study, the expression of transgenes was used to verify the influence of *yti*B and *yth*A on the capacity of bacteria to remove uranium from solutions. To the best of our knowledge, no data on the involvement of *yti*B and *yth*A genes in uranium removal has been published to date. The *yti*B gene encodes the carbonic anhydrase that catalyzes decomposition of carbonic acid into carbon dioxide and water (HCO^3−^ + H^+^ → CO_2_ + H_2_O) to help maintaining the pH balance of the cell [28]. The *yth*A gene encodes cytochrome bd coenzyme oxidase that can be found in some kinds of azotobacters, and this coenzyme has the ability to remove or reduce oxygen as part of the electron transport chain. We have found that the expression of *yti*B and *yth*A genes in the Ua strain was clearly suppressed after addition of even low concentrations of U(VI) to the growth media. There is obviously a negative correlation between the amount of *yti*B and *yth*A gene products in bacteria and the concentration of the uranium in the bacterial growth media. It is therefore reasonable to speculate that suppression of *yti*B and *yth*A gene products with U(VI) negatively influenced the condition of the bacteria, making them less tolerant to stresses, including high concentrations of uranium, and, consequently, reduced their capacity to remove U(VI) from the media.

In this study, using transcriptome sequencing we were able to confirm that the previously identified *yti*B and *yth*A genes are present and expressed only in the Ua strain of *Bacillus atrophaeus*. Using transgenic technology, we introduced *yti*B and *yth*A genes independently to the ATCC 9372 strain and demonstrated that the resulting phenotypes of the transgenic strains became very similar to the phenotype of the Ua strain; their capacity for uranium removal was reduced compared to the original wild type ATCC 9372 strain. Further investigations based on information collected by RNA sequencing should be focused on exploring the function of the genes, the regulation of their transcription by U(VI), and their roles in the molecular mechanisms of uranium removal by *Bacillus atrophaeus*.

## 3. Materials and Methods

### 3.1. Microorganisms

*Bacillus atrophaeus* ATCC 9372 strain was purchased from the Chinese General Microbiological Culture Collection (CGMCC) center. *Bacillus atrophaeus* Ua strain was isolated from a chromium mine by culturing in LB medium with 100 mg L^−1^U(VI). This strain is deposited into the CGMCC center under the strain number CGMCC 16080.

### 3.2. Analysis of U(VI) Tolerance and Removal Capacities of Bacterial Strains

ATCC 9372 and Ua strains were inoculated into 30 mL of TGY liquid medium (glucose 1.0 g L^−1^, tryptone 1.0 g L^−1^, yeast powder 3.0 g L^−1^, pH 7.0–7.2), and incubated at 37 °C for 18 h with constant shaking at 120 rpm. The OD_600_ value of each cell culture was adjusted to 0.8 with cell-free water, and then 10 mL of the inoculum was added to TGY liquid medium (90 mL) containing either 0 mg L^−1^, 40 mg L^−1^, 80 mg L^−1^, or 120 mg L^−1^ U (VI), respectively, and incubated at 37 °C for 60 h with constant shaking at 120 rpm. All experimental treatments were run in triplicate. After a 10 min centrifugation at 10,000 rpm, the OD_600_ values of each medium and the U(VI) concentration in the supernatant, were measured after 0 h, 12 h, 24 h, 36 h, 48 h, and 60 h of bacterial growth. Medium with the same U(VI) concentration, but without inoculum, was used as a negative control. The OD_600_ values were used to characterize the abilities of strains to tolerate U(VI), while U(VI) concentrations in the supernatant of media containing 120 mg L^−1^ U(VI) were used to indicate the removal capacities of strains.

Content of U (VI) was determined by the arsenazo (III) staining method [29]. 1 mL of sample was added to 1 mL of arsenazo (III) chromogenic agent, the volume of the mix was adjusted to 10 mL with 0.4 mol/L chloroacetic acid-sodium chloroacetate buffer, then the mix was shaken and left to incubate at room temperature for 30 min. Absorbance at 652 nm was measured using a spectrophotometer and microbial medium was used as control.

### 3.3. Total RNA Extraction and Transcriptome Sequencing

Bacterial cultures were grown for 48h after inoculation in 2 mL of the TGY media containing 0 and 40 mg/L uranium respectively. Total RNA was isolated from the ATCC 9372 and Ua strains using the RNAiso Plus Kit (Takara, Kyoto, Japan) according to the manufacturer’s protocol. The quantities and integrities of RNAs were measured using Qubit and Agilent 2100 (ThermoFisher, Waltham, MA, USA). Transcriptome sequencing was carried out by Illumina HiSeq 2500 (Illumina, San Diego, CA, USA) according to the manufacturer’s instructions. The adapters, low quality bases, and unknown bases were removed. The remaining clean reads were assembled using SeqPrep and Sickle software (https://github.com/jstjohn/SeqPrep; https://github.com/najoshi/sickle). Unigenes from the de novo assembly were used for bioinformatics analysis. Unigenes were annotated using Nr (non-redundant protein sequences), Nt (non-redundant nucleotides), Pfam (Protein family), Swiss-Prot, GO (Gene Ontology database), Clusters of Orthologous Groups (COG) and KEGG (Kyoto Encyclopedia of Genes and Genomes) databases. Differentially expressed genes were analyzed using RSEM software (http://deweylab.biostat.wisc.edu/rsem/).

### 3.4. Bioinformatic Analysis of ytiB and ythA Genes

DNA traces were assembled with the Phred/Phrap/Consed package (http://www.phrap.org). Sequence similarity analysis was performed using the BLAST program (http://www.ncbi.nlm.nih.gov/blast). The open reading frames (ORF) were acquired with the help of the ORF Finder tool (http://www.ncbi.nlm.nih.gov/gorf/). Protein domains were determined by Interproscan (http://www.ebi.ac.uk/interpro/) and signal peptides were identified by SignalP (http://www.cbs.dtu.dk/services/SignalP/). The secondary structures of proteins were analyzed by SOPMA (https://npsa-prabi.ibcp.fr/) and tertiary structures by SWISS-MODEL (http://www.expasy.ch/swissmod/SWISS-MODEL.html). Multiple sequence alignments were generated using Clustal W and phylogenetic trees constructed using the neighbor-joining method and the Mega 5.1 package. The reliability of the neighbor-joining tree was estimated by bootstrap analysis with 1000 repeats.

### 3.5. Analysis of the Roles of ytiB and ythA in U(VI) Removal Using a Transgenic Approach

In order to study the relationship between *yti*B and *yth*A genes and the U(VI) removal capacity, the genes were transformed into the *Bacillus atrophaeus* ATCC 9372 strain. The primers used to amplify *yth*A were: F: CGACGCGTGTGGATGATTTAGTTTTGG, R: GCTCTAGATTACGACTCCGCTGTATTTA, and for *yti*B were: F: CGACGCGTAGGGACAACGAACATGAGTCT, R: GCTCTAGAAGTCGATCCCGTTCCTGAAA. Initially, *yti*B and *yth*A were cloned into the pEASY-T1 vector using *E. coli* DH5α competent cells. Blue-White Screening was used to select transgenic *E. coli* DH5α colonies. The recombinant plasmids were isolated from the selected transgenic *E. coli* DH5α strains and digested with *Mlu*I and *Xba*I endonucleases. Fragments containing *yti*B and *yth*A genes were detected by electrophoresis and the corresponding bands were purified from the Agarose gel. The purified DNA fragments were ligated into the *Mlu*I and *Xba*I restriction sites of the recombinant pBE-S DNA expression vector. The resulting plasmids and empty vectors were transformed into the *Bacillus atrophaeus* ATCC 9372 strain, which did not contain *yti*B and *yth*A genes. Ampicillin was used for the selection of transformed cells. The transgenic strain expressing the *yti*B transgene was designated as ATCC 9372-*yti*B strain, and the transgenic strain expressing *yth*A transgene was named as ATCC 9372-*yth*A strain. The transgenic strain containing the empty pBE-S DNA vector was designated as ATCC 9372-S and used as the control. The tolerance of transgenic strains to high concentrations of U(VI) as well as their U(VI) removal capacities were evaluated as described previously. *T*-test was used to analyze the significant differences between two data.

## Figures and Tables

**Table 1:** The ratio statistics of Mapping.

Group	Number of Clean Reads	Similarity (%)
ATCC 9372-0	22534064/28206904	79.89%
ATCC 9372-U	19228202/24475060	78.56%
Ua-0	30370551/30654342	99.10%
Ua-U	39516637/40055216	98.70%

**Table 2:** The expression characteristics of *yti*B and *yth*A genes.

Gene ID	String_Tophit_Description	KEGG_Gene_Name	Length	String_topHSP_%-Simil	NR_tophit_Description	Ua_0 Count	Ua_U Count	Up- or Down-Regulation
2626	*Yti*B	*Cyn*T	570	95.72	carbonic anhydrase	294	281	down
2629	*Yth*A	*Cyd*A	1332	97.26	cytochrome D ubiquinol oxidase subunit I	350	256	down
